# Socioeconomic Status and the Sense of Coherence among Japanese People Living with HIV

**DOI:** 10.3390/ijerph19137673

**Published:** 2022-06-23

**Authors:** Taisuke Togari, Yoji Inoue, Gaku Oshima, Sakurako Abe, Rikuya Hosokawa, Yosuke Takaku

**Affiliations:** 1 School of Graduate Studies, The Open University of Japan, Chiba 261-8586, Japan; 2 Faculty of Liberal Arts, The Open University of Japan, Chiba 261-8586, Japan; yoji2006jp@yahoo.co.jp; 3 School of Information and Communication, Meiji University, Tokyo 101-8301, Japan; rescuelon@gmail.com; 4 Health Management Office, TIS Inc., Tokyo 160-0023, Japan; abe.sakurako.916@tis.co.jp; 5 Graduate School of Medicine, Kyoto University, Kyoto 606-8507, Japan; hosokawa.rikuya.4r@kyoto-u.ac.jp; 6 Japanese Network of People Living with HIV/AIDS, Tokyo 169-0073, Japan; ytakaku@janpplus.jp

**Keywords:** HIV/AIDS, sense of coherence, socioeconomic status, Japanese

## Abstract

People living with HIV (PLWH) are forced to live with multiple and severe stressors. Focusing on sense of coherence (SOC), which is a concept of salutogenic and stress coping capacity, is useful in PLWH support. This study aimed to examine the association between SOC and socioeconomic status (SES) for Japanese PLWH. Methods: This study used data from the HIV Futures Japan national survey, which is an online survey with a cross-sectional design. This survey of PLWH in Japan was conducted from July 2013 to February 2014 and December 2016 to July 2017, resulting in 1422 valid responses. The mean age (SD) was 38.6 years (8.3). The 13-item SOC scale score was divided into two groups, based on Japanese standard score in a previous study, and logistic regression analysis was performed. Results: Education levels were indirectly associated with SOC through occupation. Compared to freelance-profession/self-employed, “unemployed job seekers” (OR [95%CI] = 2.16 [1.16, 4.04]) and “homemaker/recuperating/student” (2.09 [1.09, 4.02]) were directly related to poor SOC, regardless of income. Also, there is a clear SOC disparity in income (compared to “>8 million yen/year”, “<1 million yen/year” was 2.94 [1.46, 5.92], and “1–2.99 million yen/year” was 2.49 [1.33, 4.68]). Conclusion: It became clear that there is a relationship between SOC and SES. The results of this research provide important evidence for health promotion measures for PLWH.

## 1. Introduction

In Japan, the number of new human immunodeficiency virus (HIV)/acquired immunodeficiency syndrome (AIDS) cases was 1095 in 2020 (1233 in 2019) [1]. The total number of reports for 1985–2020 was 32,461 (including those who subsequently died) [1]. Although the number of new reports per year has been declining in recent years, the number of HIV/AIDS patients is still increasing annually in Japan.

A study has reported that over 70% of individuals living with HIV in Japan have some sort of anxiety or stress [2], and the biggest psychosocial stressor for people living with HIV (PLWH) is said to be the stigma [3,4]. In Japan, 86.9% of PLWH have internalized stigma [5]. As a result of these circumstances, 41.7% of individuals living with HIV in Japan have some form of mental health problem [5] and a significantly higher proportion of PLWH have depressive tendencies or anxious tendencies, in comparison to the general population [2,5].

Aaron Antonovsky, one of the pioneers in the field of health promotion, argued for the need for Salutogenesis [6]. Salutogenesis is a perspective under the question “What creates health?”, that advocates seeking out and promoting salutary factors [7], and is in an oppositional perspective to pathogenesis, which seeks the cause of disease and risk-factors [6]. Based on the theory of Salutogenesis, Antonovsky presented the salutogenic model that modeled the process leading to health generation by integrating various previous studies and social science theories. The salutogenic model is based on stress theory and the premise that “ubiquitous stressor” exists in the world of human beings, and includes cultural-historical factors, socioeconomic factors, psychosocial factors, and biological factors contributing to the success of coping with ubiquitous stressors [6,8]. It is a model that leads to health through neuro-physiological processes [6,8]. The sense of coherence is a core concept of the salutogenic model. A sense of coherence is a concept related to a way of viewing and dealing with life that involves successfully managing stressors, thus leading to health. The complete definition is as follows:

*The sense of coherence is a global orientation that expresses the extent to which one has a pervasive, enduring though dynamic feeling of confidence that (1) the stimuli deriving from one’s internal and external environments in the course of living are structured, predictable, and explicable (comprehensibility); (2) the resources are available to one to meet the demands posed by these stimuli (manageability); and (3) these demands are challenges, worthy of investment and engagement (meaningfulness)*. [9]

According to the salutogenic model [9], in a person with a strong sense of coherence, a variety of general resistance resources (GRRs) are mobilized to adroitly and readily deal with various stressors in life; this leads to successful coping and it helps to maintain and promote health. All of the resources (people, things, money, knowledge, wisdom, social relationships, socioeconomic status, genetic factors, etc.) that a person can draw on to resist stressors are referred to as GRRs. GRRs are not only mobilized in dealing with stressors, but also represent a group of elements that provide the good life experiences that shape the sense of coherence [9]. Also, according to Antonovsky, work content is deeply related to good life experience [9]. Occupations that require complex, sophisticated techniques, high decision latitudes, and are stable are more likely to produce a high sense of coherence. However, it is also hypothesized that occupations with lower decision latitudes and higher job insecurity will produce a lower sense of coherence [9].

Reliable and valid scales have been developed to gauge a sense of coherence [10]. Furthermore, sense of coherence is closely associated with health [11]. Therefore, salutogenesis and sense of coherence play a major role in policy making and evaluation of stress coping, stressor reduction and health promotion.

However, it has been repeatedly reported that the sense of coherence in Japanese PLWH has a statistically significant lower score than the general population [5,12,13]. According to previous reports in Sweden, PLWH have a lower sense of coherence than that of the general population [14]. Therefore, it is significant for health promotion in PLWH to clarify their sense of coherence and the social determinants of their sense of coherence.

Previous studies on the general population revealed that individuals with more years of education had a higher sense of coherence [15,16,17,18,19,20,21], and that unemployed individuals, and individuals receiving a disability pension, had a lower sense of coherence than individuals who were employed [15,16,17,21]. A follow-up study of the association between one’s sense of coherence and current financial situation found that women with a lower household income had a lower sense of coherence, but a higher income did not result in a higher sense of coherence [22]. Another study found no association between the level of one’s personal income and the level of one’s sense of coherence [23]. Yet another study reported that one’s score on a sense of coherence scale increased as one’s level of deprivation decreased [17]. A further Japanese study reported that women with a higher household income had a higher sense of coherence [21].

However, socioeconomic status affecting a sense of coherence has not been examined in PLWH. In Japan, education level and occupation are closely related, and occupation and income are also closely related [24]. The current study used national survey data to examine the sense of coherence in Japanese men and women living with HIV. The aim of this study was to verify the following model for Japanese PLWH. That is, low education, those with high job insecurity and the unemployed, and low annual income are associated with a low sense of coherence. At the same time, we examined these models in a multivariate analysis to consider the interrelationships of education, employment status and income.

## 2. Materials and Methods

### 2.1. Subjects and Methods

This cross-sectional study employed data from the first and second HIV Futures Japan (HFJ) nationwide surveys (HFJ-wave1 and HFJ-wave2) of Japanese people living with HIV (PLWH).

Three authors of this paper, including the corresponding author, are members of the HFJ steering committee. A participatory research method was used to implement the survey; advertising and recruiting were achieved through support groups for PLWH, a public social network service account (e.g., Twitter, Facebook, etc.), and 400 or more HIV treatment institutions across Japan. Participatory research is the co-construction of studies among researchers, people affected by the issues under study (e.g., patients, community members), and/or decision makers who apply research findings (e.g., health managers, policymakers, and community leaders) [25,26,27]. The composition of the HIV Futures Japan project team is as follows. The “steering group (two researchers, two PLWH)” supervises the project. The “researcher group (fifteen researchers)” formulates research protocols, questionnaires, and analyzes data. The “reference group (fifteen PLWH)” checks the survey method, survey form content, and analysis content and adds comments. The “reference group” is a representative of PLWH peer support groups in various regions throughout Japan. We promoted the survey and encouraged participation in the survey for PLWH. Under the leadership of the steering group, each group cooperated and the project was promoted.

**Figure 1:**
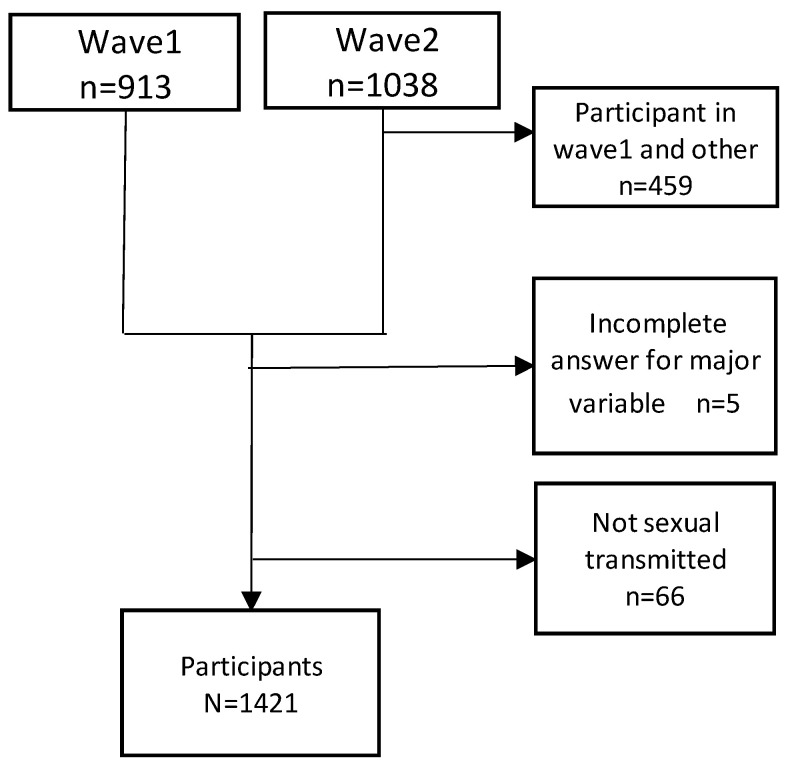
Flow chart of participants in this study.

A flow chart of the participants in this study is shown in Figure 1. HFJ-wave1 was an anonymous self-administered Web survey conducted from July 2013 to February 2014. Responses were obtained from 1095 people; after data cleaning, 913 valid responses remained. HFJ-wave2 was implemented from December 2016 to July 2017, using the same survey format as that for HFJ-wave1; 1110 individuals responded, resulting in 1038 valid responses. Thereafter, data cleaning was performed to remove cases where there were few answers, or cases where the answers were incomplete, such as cases where all the answer numbers were the same.

The results from 579 individuals who responded to HFJ-wave2, but did not respond to HFJ-wave1, were combined with the 913 valid responses from HFJ-wave1. We set the following three criteria for exclusion from HFJ data. First, wave2 respondents who answered wave1 were excluded. Second, persons who do not have answers for the main attribute variables were excluded. Lastly, people who had a transmission route other than sexually transmitted infection were excluded. The latter exclusion was because most of these respondents are victims of phytotoxicity, and their socio-economic situation is very different from those who have sexually transmitted infections. As these surveys were open entry online surveys, the recovery rate could not be calculated. We then analyzed the data from 1422 individuals. A flowchart of the sample appears in Figure 1. The mean age (SD) was 38.6 (8.3) years.

In requesting research cooperation, we described the following on the web page and provided sufficient explanation; research subject, research purpose, voluntary subject, expected advantages and disadvantages, strict protection of personal information, maintenance of anonymity, no disadvantage in case of non-participation, protection of human rights. We obtained informed consent before conducting the survey.

In conducting the web survey, the following consideration was given, so that personal information would not be identified. IP addresses were recorded to prevent unauthorized access and avoid duplicate answers, but this was strictly centralized by the administrator. Answer data was encrypted by Secure Sockets Layer and sent. When disclosing the research results, anonymization was ensured, so that individuals and specific facilities were not identified, and privacy was thoroughly protected.

All research procedures were in accordance with the “Declaration of Helsinki” and “Ethical Guidelines for Medical and Health Research Involving Human Subjects”.

### 2.2. Variables

#### 2.2.1. Sense of Coherence

The current study used the 13-item version of the sense of coherence scale (SOC-13) [9,10]. Item examples were “Do you have the feeling that you are in an unfamiliar situation and don’t know what to do?”, and “How often do you have feelings that you’re not sure you can keep under control?”. Responses to each item were selected on a 7-point semantic differential scale. The range of scores was 13–91 points. The higher the SOC-13 score, the higher the stress coping capacity. The reliability and validity of the Japanese version had also been verified [28]. In the current study, Cronbach’s α was 0.83. Comparison of SOC scores between the sample of this study and the Japanese national representative sample, by age group, are shown in Appendix A. In this study sample as well, the SOC score was significantly lower than that of the Japanese general population sample in any age group under 55 years old.

In this study, SOC-13 was divided into high, medium, and low using the data of the national representative sample [29], instead of the SOC-13 total score. Basing the set cutoff value on the distribution of this survey sample (specific sample) was an advantage because the group size could be adjusted in the form of median, tertile and quartile, etc. The risk of cutting off the use of country sample data was that the group size could not be adjusted. On the other hand, the relative classification, based on the specific sample, might be incorporated into a moderate group despite, for instance, low levels of real SOC function. This brought about a departure from the concept of high and low based on the standard score. Based on the above, we decided to use the standard values of the national sample data.

Specifically, the group with a Japanese mean +1SD and greater score was designated as a high SOC group, the group with a Japanese mean −1SD or lesser score was designated as a low SOC group, and the other intermediate groups (Table 1). The reasons for this grouping were as follows. First, when cutting off at the median, there is a risk that the definition of good/poor is ambiguous because the score around the median is included in either. Second, by setting ±1SD as the reference interval and positioning the middle layer, the definition of good/poor in the group became clearer. Third, clinically targeting a group with low stress coping capacity was a better way for obtaining clues for support.

#### 2.2.2. Socioeconomic Status

Level of education: Respondents were asked to “Please indicate the highest level of education you received”. Considering the answers, responses were classified into three categories: “middle/high school”, “vocational school/junior college/technical school”, and “university/graduate school”.

Working status: One’s current type of employment, profession, and lack of employment were classified into a total of nine categories. Six categories were used to describe respondents who were working: “freelance profession/self-employed”, management/executive”, “full-time employee”, “temporary or contract employee”, “part-time or temporary agency employee”, and “some other type of work”. Three categories were used to describe respondents who were unemployed: “looking for a job”, “homemaker/recuperating from an illness/student”, and “worklessness (and not falling into the other 2 categories)”.

Personal income: Annual personal income in the previous year was classified into the following five categories: “less than 1 million yen”, “1 million to 3 million yen”, “3 million to 5 million yen”, “5 million to 8 million yen”, and “more than 8 million yen”.

#### 2.2.3. Control Variables

Gender and age: Gender were divided into three categories: female, male, and other. We obtained the answer of the actual age at the time of the survey.

Years since testing HIV-positive: Respondents were asked “When did you learn that you were infected with HIV” and they were asked to respond with the specific year. This value was converted to the number of years prior to the survey. Based on the distribution of this value, responses were classified into the following three categories: “less than 3 years”, “4 to 9 years”, and “over 10 years”.

### 2.3. Statistical Analysis

Based on the data distribution and theoretical meaning, we decided to treat the data as a binary variable related to a low sense of coherence group and a middle to high sense of coherence group. Logistic regression analysis was performed with sense of coherence as a binary variable, with the dependent variable adjusted for gender, age, and years since testing HIV-positive. We performed a hierarchical regression analysis to examine the relationships between socioeconomic status; model 1 had education level as the independent variable, model 2 had education level and working status as independent variables, and model 3 had education level, working status and personal annual income as independent variables. The above analyses were performed using IBM SPSS statistics 28, and the level of statistical significance was 5%.

## 3. Results

### 3.1. Descriptive Statistics of Demographic Variables and Socioeconomic Status by Sense of Coherence

The frequency distribution of each variable by the sense of coherence is shown in Table 2. There was no association between gender and years since testing HIV-positive and the sense of coherence levels. There was a significant association for age (Cramér’s V = 0.095, *p* = 0.047).

### 3.2. Relationships between Socioeconomic Status and Low Sense of Coherence

Relationships between socioeconomic status and low sense of coherence are shown in Table 3. Regarding the level of education, in model1, the odds ratio [95% confidence interval: lower limit, upper limit] of “middle/high school” was 1.33 [1.04, 1.70], compared to “university/graduate school”. However, no association was found in model 2 or 3.

Regarding working status, in model 2, the odds ratio of “fixed-term or contract employee” was 1.93 [1.10, 3.39], compared to “freelance profession/self-employed”. Of the unemployment formats, “looking for a job” was 2.63 [1.43, 4.86], “homemaker/recuperating from an illness/student” was 2.63 [1.42, 4.87], and “worklessness” was 2.10 [1.08, 4.09]. In Model 3, “looking for a job” was 2.16 [1.16, 4.04] and “homemaker/recuperating from an illness/student” was 2.09 [1.09, 4.02].

Regarding personal annual income, the odds ratio for the group of “1 to 2.99 million yen” (about 0.8 to 2.5 million euro) was 2.49 [1.33, 4.68], compared to the group of “8 million yen (about 6.7 million euro) or more. The odds ratio for the group below 1 million yen was 2.94 [1.46, 5.92].

## 4. Discussion

### 4.1. Sense of Coherence and Socioeconomic Status

Examination of the association between the sense of coherence and socioeconomic status yielded the following findings. It became clear that the relationship between the sense of coherence and socioeconomic status, which consisted of education level, occupation level, and income, was greater than in previous studies, in terms of statistical significance. Although there was a difference between the analysis method of this research, which performed logistic regression analysis, while the analysis method of the previous research was based on linear regression.

One of the reasons for this difference in results was that the sense of coherence was used as a binary variable in this study. In other words, it is highly possible that the relationship between socioeconomic status and the sense of coherence is not a linear relationship. Also, the sense of coherence in PLWH is more vulnerable to social environmental damage than the general population. Further investigation is required on this point, including its mechanism.

Education levels were indirectly, rather than directly, associated with sense of coherence through occupation. Previous studies in the general population have reported direct effects [20]. There are also reports that education has a direct effect on the sense of coherence by adjusting occupations without controlling income [15]. According to the distribution of education levels in the subjects of this study, the ratio of “university/graduate school” graduates was 46.9%. In the Japanese population census in 2015, the percentage of those who graduated from university or graduate school was 19.9%, and at this time, the percentage of those with a high educational background was high. Therefore, the results of this study may not fully show the effect of education level.

It was shown that “unemployed job seekers” and “homemaker/recuperating from an illness/student” were directly related, regardless of income. This finding is similar to the results of previous studies in the general population [15,16,17,21]. There are very few previous studies that controlled income and showed the same association, and only reports on the longitudinal design in the Japanese general sample [21]. Occupation and income may be closely related among Japanese people. However, it is necessary to examine the reproducibility of similar models in samples from other countries. Also, there is a special reason for this PLWH sample. The age group of people who are “homemaker/recuperating from an illness/student” is 96.3% for those aged 25 and over, and 12.2% for women. Therefore, many of them are people who do not work and concentrate on the treatment of their illness. The inability to lead a professional life with the illness of HIV/AIDS has become an extremely large stressor. On the contrary, having a job and having vocational ability are powerful general resistance resources.

There was a clear disparity in income. Most previous studies did not show the association [22,23]. However, the results of a large study of women raising children in four Scandinavian countries clearly showed the association [20]. Also, a survey of the general Japanese population showed these relationships in women [21]. This study showed that there was a clear disparity in sense of coherence in the income class according to the hypothesis based on the salutogenic model. This study showed gender-adjusted results, but 96% were male. In addition, since the influence of working status was attenuated by adding income to the regression equation, income was a mediator of occupational influence. Therefore, it can be said that income is an extremely powerful general resistance resource in PLWH. In addition, the large income gap in sense of coherence means that low-income PLWH will often fail to deal with stress in the future. There are concerns about the future health deterioration of low-income PLWH, as the sense of coherence has been shown, by several long-term cohort studies, to predict health status.

On the other hand, it is undeniable that the level of the sense of coherence may define SES, because the sense of coherence is a stress coping ability. In other words, it is possible to hypothesize that the higher the sense of coherence, the easier it is to achieve status. In order to test this hypothesis, it is necessary to study with a longitudinal design. Further research is needed in the future.

### 4.2. Implications

SES is one of the social determinants of health in all people [30]. Since the sense of coherence has been shown to be predictive of health status, a theoretical implication is that the sense of coherence is a mediator in the effects of SES on health outcomes. Although there are some epidemiological findings on the sense of coherence and its effects on health outcomes, including psychological distress, the details of health-affecting mechanisms, including the functioning of the sense of coherence components, have not been fully demonstrated. Further studies are needed in the future.

Next, it is necessary to develop a program that is expected to improve the sense of coherence and apply the program to PLWH with low SES. In order to improve the sense of coherence, it is important to positively recognize a good life experience. It will be possible to develop a program specialized for the life world of PLWH.

Finally, the importance of conducting an assessment for SES to support the mental health and psychological well-being of PLWH is clear. Since PLWH with a low SES are likely to be vulnerable to stress coping, it is necessary to focus on them in a stress coping support approach.

### 4.3. Limitations

This study was the first to clarify the relationship between socioeconomic status and sense of coherence in PLWH, but it has the following limitations. First, this study design was a cross-sectional study, and the causal relationship between socioeconomic status and sense of coherence was not clarified. It is theoretically possible that low income is due to low sense of coherence. At the same time, it is possible that socioeconomic status and sense of coherence have a spiral relationship. In the future, it is necessary to carefully consider the relationship in a longitudinal design.

Second, the current study was an open online survey. This study was not conducted by medical personnel, such as physicians, so subjects included individuals who tested HIV-positive but who were not visiting a medical facility for treatment. This was an advantage, but the resulting sample composed mostly younger individuals who were used to online surveys. In the future, relatively older age groups need to be surveyed.

Last, socioeconomic status responses were self-reported. Respondents’ responses may have overvalued the levels of the indices. In the future, reproducibility will need to be validated based on objective social indicators.

## 5. Conclusions

The aim of the current study was to use data on Japanese people living with HIV from a national survey to examine the association between a sense of coherence and socioeconomic status. As a result, the following four points were clarified. First, it became clear that the relationship between the sense of coherence and socioeconomic status, which consists of education level, occupation level, and income, is greater than in previous studies, in terms of statistical significance. Second, education levels were indirectly, rather than directly, associated with sense of coherence through occupation. Third, it was shown that unemployed job seekers and people in the “homemaker/recuperating from an illness/student” group were directly related, regardless of income. Finally, there was a clear disparity in income.

## Figures and Tables

**Table 1:** Sense of coherence classification in this study based on Japanese representative sample.

	*n*	Mean	(SD)
Low group (less than 46.8: standard score * −1SD)	495	37.5	(6.9)
Middle group (46.8 points or more and less than 71.2 points)	823	56.2	(6.5)
High group (71.2 points or more: stangard score +1SD)	103	78.1	(5.1)

* Mean sense of coherence score from a Japanese representative sample [29].

**Table 2:** Sample statistics of demographic variables and socioeconomic status by low and middle to high sense of coherence.

	Mid-High SOC (*n* = 926)		Low SOC (*n* = 495)		Total (*n* = 1421)		*p* *
	n	(%)	n	(%)	n	(%)	
Gender							0.371
Female	30	(3.2)	11	(2.2)	41	(2.9)	
Male	892	(96.3)	480	(97.0)	1373	(96.6)	
Other	4	(0.4)	4	(0.8)	8	(0.6)	
Age group							0.047
–24	26	(2.8)	16	(3.2)	42	(3.0)	
25–34	275	(29.7)	158	(31.9)	433	(30.5)	
35–44	398	(43.0)	225	(45.5)	623	(43.8)	
45–54	183	(19.8)	88	(17.8)	271	(19.1)	
55–64	35	(3.8)	5	(1.0)	40	(2.8)	
65–	4	(0.4)	0	(0.0)	4	(0.3)	
no answer	5	(0.5)	3	(0.6)	8	(0.6)	
Years since testing HIV-positive							0.895
less than 3years	411	(44.4)	226	(45.7)	637	(44.8)	
4–9 years	337	(36.4)	177	(35.8)	514	(36.2)	
over 10 years	178	(19.2)	92	(18.6)	270	(19.0)	
Education							0.059
middle/high school	305	(32.9)	188	(38.0)	493	(34.7)	
vocational school/junior college/technical school	166	(17.9)	96	(19.4)	262	(18.4)	
university/graduate school	455	(49.1)	211	(42.6)	666	(46.9)	
Working status							<0.001
Working formats							
freelance profession/self-employed	83	(9.0)	29	(5.9)	112	(7.9)	
management/executive	35	(3.8)	14	(2.8)	49	(3.4)	
full-time employee	480	(51.8)	212	(42.8)	692	(48.7)	
fixed-term or contract employee	73	(7.9)	51	(10.3)	124	(8.7)	
part-time or temporary agency employee	125	(13.5)	77	(15.6)	202	(14.2)	
some other type of work	8	(0.9)	6	(1.2)	14	(1.0)	
Unemployment formats							
looking for a job	43	(4.6)	41	(8.3)	84	(5.9)	
homemaker/recuperating from an illness/student	43	(4.6)	39	(7.9)	82	(5.8)	
Worklessness	36	(3.9)	26	(5.3)	62	(4.4)	
Personal annual income (in yen)							<0.001
<1 million	111	(12.0)	97	(19.6)	208	(14.6)	
1–2.99 million	259	(28.0)	178	(36.0)	437	(30.8)	
3–4.99 million	300	(32.4)	137	(27.7)	437	(30.8)	
5–7.99 million	167	(18.0)	57	(11.5)	224	(15.8)	
≥8 million	70	(7.6)	16	(3.2)	86	(6.1)	
no answer	19	(2.1)	10	(2.0)	29	(2.0)	

SOC: sense of coherence. * chi-square test.

**Table 3:** Logistic regression analysis about relationships between socioeconomic status and low sense of coherence among Japanese people living with HIV/AIDS.

	Univariate			Model 1			Model 2			Model 3		
	OR	95%CI	*p*	OR	95%CI	*p*	OR	95%CI	*p*	OR	95%CI	*p*
		[LL, UL]			[LL, UL]			[LL, UL]			[LL, UL]	
Level of education												
middle/high school	1.33	[1.04, 1.70]	0.022	1.33	[1.04, 1.70]	0.026	1.14	[0.88, 1.48]	0.326	1.04	[0.80, 1.36]	0.758
vocational school/junior college/technical school	1.25	[0.92, 1.68]	0.149	1.23	[0.91, 1.66]	0.187	1.16	[0.85, 1.58]	0.347	1.04	[0.76, 1.43]	0.789
university/graduate school	1.00			1.00			1.00			1.00		
Working status												
freelance profession/self-employed	1.00						1.00			1.00		
management/executive	1.15	[0.54, 2.42]	0.724				1.16	[0.55, 2.48]	0.698	1.39	[0.65, 3.01]	0.399
full-time employee	1.26	[0.80, 1.99]	0.310				1.22	[0.77, 1.94]	0.392	1.43	[0.89, 2.30]	0.136
fixed-term or contract employee	2.00	[1.15, 3.48]	0.014				1.93	[1.10, 3.39]	0.021	1.70	[0.96, 3.02]	0.068
part-time or temporary agency employee	1.76	[1.06, 2.93]	0.029				1.59	[0.94, 2.68]	0.082	1.32	[0.78, 2.24]	0.309
some other type of work	2.15	[0.69, 6.71]	0.189				2.02	[0.64, 6.41]	0.231	1.81	[0.57, 5.78]	0.317
looking for a job	2.73	[1.50, 4.98]	0.001				2.63	[1.43, 4.86]	0.002	2.16	[1.16, 4.04]	0.016
homemaker/recuperating from an illness/student	2.60	[1.42, 4.76]	0.002				2.63	[1.42, 4.87]	0.002	2.09	[1.09, 4.02]	0.027
worklessness	2.07	[1.07, 3.99]	0.031				2.10	[1.08, 4.09]	0.030	1.60	[0.79, 3.23]	0.189
Personal annual income (in yen)												
<1 million	3.82	[2.08, 7.02]	<0.001							2.94	[1.46, 5.92]	0.003
1–2.99 million	3.01	[1.69, 5.35]	<0.001							2.49	[1.33, 4.68]	0.005
3–4.99 million	2.00	[1.12, 3.57]	0.019							1.71	[0.94, 3.11]	0.082
5–7.99 million	1.49	[0.80, 2.78]	0.206							1.35	[0.72, 2.53]	0.353
≥8 million	1.00									1.00		
no answer	2.30	[0.90, 5.89]	0.082							1.63	[0.59, 4.51]	0.350
−2 loglikelihood				1808.86			1783.75			1766.84		
Nagelkerke R^2^				0.017			0.041			0.057		

OR: odds ratio, CI: confidential interval, LL: lower confidence limit, UL: upper confidence limit. Models 1 to 3 were adjusted for gender, age years, and years since testing HIV-positive. Dependent binary variable was “low sense of coherence” = 1.

**Figure A1:**
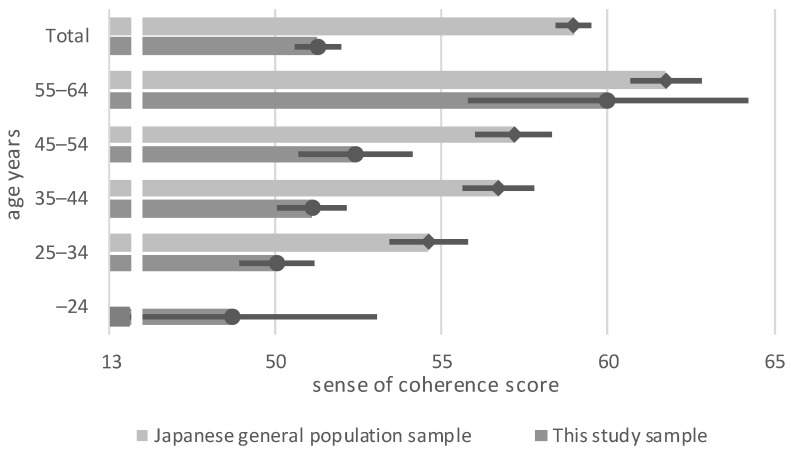
Comparison of the sense of coherence scores between the sample of this study and the Japanese general population sample by age group. Note. Error bars are 95% confidential interval.

## Data Availability

The data presented in this study are available on request from the corresponding author. The data are not publicly available due to patients’ privacy.
